# Cortical abnormalities of synaptic vesicle protein 2A in focal cortical dysplasia type II identified in vivo with ^18^F-SynVesT-1 positron emission tomography imaging

**DOI:** 10.1007/s00259-021-05665-w

**Published:** 2022-01-03

**Authors:** Yongxiang Tang, Jie Yu, Ming Zhou, Jian Li, Tingting Long, Yulai Li, Li Feng, Dengming Chen, Zhiquan Yang, Yiyun Huang, Shuo Hu

**Affiliations:** 1 Department of Nuclear Medicine, Xiangya Hospital, Central South University, 87 Xiangya Road, Changsha, 410008 Hunan China; 2 Department of Neurology, Xiangya Hospital, Central South University, Changsha, Hunan China; 3 Department of Neurosurgery, Xiangya Hospital, Central South University, Changsha, Hunan China; 4 PET Center, Department of Radiology and Biomedical Imaging, Yale University School of Medicine, 801 Howard Ave, P.O. Box 208048, New Haven, CT 06520-8048 USA; 5 Key Laboratory of Biological Nanotechnology of National Health Commission, Xiangya Hospital, Central South University, Changsha, Hunan China; 6 National Clinical Research Center for Geriatric Disorders (Xiangya), Xiangya Hospital, Central South University, Changsha, Hunan China

**Keywords:** Focal cortical dysplasia, PET, SV2A, FDG, Epilepsy

## Abstract

**Purpose:**

The loss of synaptic vesicle glycoprotein 2A (SV2A) is well established as the major correlate of epileptogenesis in focal cortical dysplasia type II (FCD II), but this has not been directly tested in vivo. In this positron emission tomography (PET) study with the new tracer ^18^F-SynVesT-1, we evaluated SV2A abnormalities in patients with FCD II and compared the pattern to ^18^F-fluorodeoxyglucose (^18^F-FDG).

**Methods:**

Sixteen patients with proven FCD II and 16 healthy controls were recruited. All FCD II patients underwent magnetic resonance imaging (MRI) and static PET imaging with both ^18^F-SynVesT-1 and ^18^F-FDG, while the controls underwent MRI and PET with only ^18^F-SynVesT-1. Visual assessment of PET images was undertaken. The standardized uptake values (SUVs) of ^18^F-SynVesT-1 were computed for regions of interest (ROIs), along with SUV ratio (SUVr) between ROI and centrum semiovale (white matter). Asymmetry indices (AIs) were analyzed between the lesion and the contralateral hemisphere for intersubject comparisons.

**Results:**

Lesions in the brains of FCD II patients had significantly reduced ^18^F-SynVesT-1 uptake compared with contralateral regions, and brains of the controls. ^18^F-SynVesT-1 PET indicated low lesion uptake in 14 patients (87.5%), corresponding to hypometabolism detected by ^18^F-FDG PET, with higher accuracy for lesion localization than MRI (43.8%) (*P* < 0.05). AI analyses demonstrated that in the lesions, SUVr for each of the radiotracers were not significantly different (*P* > 0.05), and ^18^F-SynVesT-1 SUVr correlated with that of ^18^F-FDG across subjects (*R*^2^ = 0.41, *P* = 0.008). Subsequent visual ratings indicated that ^18^F-SynVesT-1 uptake had a more restricted pattern of reduction than ^18^F-FDG uptake in FCD II lesions (*P* < 0.05).

**Conclusion:**

SV2A PET with ^18^F-SynVesT-1 shows a higher accuracy for the localization of FCD II lesions than MRI and a more restricted pattern of abnormality than ^18^F-FDG PET.

**Supplementary Information:**

The online version contains supplementary material available at 10.1007/s00259-021-05665-w.

## Introduction

Focal cortical dysplasia type II (FCD II) constitutes the most common cause of seizures in patients who undergo surgery before the age of 18 years [1]. Epilepsy in FCD II is commonly pharmacoresistant and thus particularly challenging for antiepileptic treatment [2]. Surgical resection of FCD II lesions may prevent seizures and improve quality of life [3]. It has been well established that the main predictor of favorable surgical outcomes is the complete removal of the dysplastic cortex.

FCD II is predominantly located in extratemporal areas, in particular the eloquent cortex [3]. Magnetic resonance imaging (MRI) features in FCD II have been widely described [4], but so-called negative MRI has been reported in 17–34% of patients and is associated with poor surgical outcomes [5]. Positron emission tomography (PET) imaging with ^18^F-fluorodeoxyglucose (^18^F-FDG) has significantly improved the positive detection rate of lesions. However, maximal hypometabolic areas correspond to both the lesion and seizure onset zone [6]. The accuracy of ^18^F-FDG PET in identifying FCD II is limited by hypometabolism frequently extending beyond the lesion. Therefore, for FCD II patients, PET imaging with additional radioligands that can be used to guide more accurate demarcation of the lesion would be of great clinical value.

Observations from a rat model of epilepsy and dysplastic cortical tissue suggested that the loss of synaptic vesicle glycoprotein 2A (SV2A) may lead to alterations in neurotransmission [7]. SV2A loss can cause impairments in γ-aminobutyric acid (GABA)ergic function [8–10]. ^11^C-UCB-J, a specific radioligand for SV2A, has been used in the investigation of several neuropsychiatric diseases [10–13]. Compared to ^11^C-UCB-J, the newly reported SV2A radioligand ^18^F-SynVesT-1 has a longer half-life and superior signal-to-noise ratio [14, 15]. In a preliminary study using static ^18^F-SynVesT-1 PET, we demonstrated lower SV2A levels in the epileptogenic zone (EZ) of patients with FCD II [16]. In the present study, we included more FCD II patients with neuropathology data and controls. The FCD II patients were also evaluated with ^18^F-FDG PET and high-resolution MRI to allow for direct comparisons.

## Materials and methods

### Participants

Sixteen FCD II patients and 16 controls were included in the present study. Localization of the EZ was determined by at least 2 experienced epileptologists based on all available clinical, video-electroencephalographic (EEG), interictal EEG, neuroimaging, and invasive stereo-EEG (SEEG) monitoring data if indicated. Sixteen consecutive patients underwent surgery for intractable epilepsy and histologically proven FCD II (FCD type II includes two subgroups based on the absence (IIa) or presence (IIb) of balloon cells) [17]. The exclusion criteria included any current or past clinically significant medical or neurological illness (other than FCD) that could have affected the study outcome. Some antiepileptic drugs (AEDs) are known to decrease cerebral blood flow and metabolism [18, 19], and levetiracetam and brivaracetam bind to SV2A [20, 21]. Patients were excluded if they were taking levetiracetam or brivaracetam. Those who could discontinue AED were instructed to withhold their medication so that their last dose was at least 24 h before the scheduled ^18^F-SynVesT-1 injection time. Other patients who could not discontinue AED administration because of seizures that were too frequent were excluded from the study. All patients were closely monitored by a neurologist during MRI and PET imaging, and no clinical seizures were noted.

The study protocol was approved by the Human Investigation Committee and Radiation Safety Committee at Xiangya Hospital, Central South University. All participants provided written informed consent prior to participating in the study.

#### MRI

All participants underwent a structural MRI scan using the 3-T Siemens MAGNETOM Trio, a Tim system. A high-resolution, 3D magnetization-prepared rapid acquisition with gradient echo (MPRAGE) T_1_-weighted sequence was used to identify structural abnormalities and for coregistration with PET images (repetition time = 2300.0 ms, echo time = 3.0 ms, field of view (FOV) = 256 × 256 mm, slice thickness = 1.0-mm thick contiguous slices, 176 sagittal slices, voxel size = 1.0 × 1.0 × 1.0 mm).

### PET imaging

^18^F-SynVesT-1 was synthesized using previously described methods [22]. Participants discontinued all AEDs for at least 24 h before PET scans and fasted for at least 6 h before ^18^F-FDG injection. Patients were monitored and confirmed to have had no clinically visible seizures within 24 h before PET examinations. Continuous EEG recording was started 2 h before radioligand injection to ensure the lack of seizure and that the radioligands were not administered in a postictal situation [23]. All patients were scanned first with ^18^F-FDG and then with ^18^F-SynVesT-1 at the same time on the following day, while controls had only an ^18^F-SynVesT-1 PET scan. Static PET images were acquired in three dimensions for 5 min, starting at ~ 60 min after intravenous injection of the radioligands. PET/computed tomography (CT) images were acquired by a Discovery Elite PET/CT scanner (GE Healthcare, Waukesha, USA). The scanning protocol was the same as described previously [24].

### Visual assessment of MRI and PET images

Visual MRI analysis was performed by two experienced neuroradiologists blinded to the clinical data. MRI was classified as positive if the images demonstrated FCD II features [4] and nonspecific or negative in the remaining cases. PET images were visually evaluated by two nuclear medicine specialists who were unaware of the clinical, EEG, and MRI findings. These visual assessments were performed to compare the accuracy of these images in locating the lesion. The 5-min frame of PET images was used to calculate the standardized uptake value (SUV) for ^18^F-FDG and ^18^F-SynVesT-1. Nuclear medicine physicians were asked to identify abnormal areas in the PET images to localize the EZ to 1 of 8 sites (left or right; frontal, temporal, parietal, or occipital) or classify them as nonlocalized. Then, PET images were coregistered to the participant’s T_1_-weighted MR image using SPM12. Individual PET/MRI images were then analyzed by comparing the localization consistency and extent of the uptake abnormalities in the lesion between PET images with the two radiotracers.

### Semiquantitative analysis of PET/MRI images

Semiquantitative analysis was performed for all PET data. SUV was calculated for all regions of interest (ROIs), and SUV ratio (SUVr) with the centrum semiovale (CS) as reference region was calculated for interpatient comparisons, as the CS has been reported to be free of SV2A and thus can be used as a reference region [10]. The asymmetry index (AI) was determined to evaluate the intensity of regional abnormalities in metabolism or ^18^F-SynVesT-1 uptake. ROIs for the lesion and contralateral region were delineated manually on both PET/MRI images by a single operator in combination with the visual assessment and pathology results [25]. AIs were calculated in control subjects as 200% × ([left − right]/[left + right]) and in patients as 100% × ([contralateral − ipsilateral]/[contralateral + ipsilateral]). The SUVr and AI for 8 major nonlesioned brain regions (left and right; frontal, temporal, parietal, or occipital) were also calculated for both patients and controls.

### Visual ratings (^18^F-FDG PET versus ^18^F-SynVesT-1 PET)

In subsequent visual ratings, two experienced nuclear medicine specialists reviewed the PET images with knowledge of clinical data and lesion localization. The reviewers were asked to grade the extent of the abnormalities in ^18^F-SynVesT-1 and ^18^F-FDG PET images by using the PET/MRI coregistered image as a reference for anatomical delineation. All lesions with abnormal ^18^F-FDG or ^18^F-SynVesT-1 uptake were scored as follows: mild, 1 = involving the focal gyrus; moderate, 2 = involving a single gyrus; severe, 3 = involving several gyri in the same lobe or several lobes and regions, similar to that described previously for the grading of ^18^F-flumazenil (FMZ) and ^18^F-FDG PET imaging results in patients with temporal lobe epilepsy [9], except that intensity was not factored in the rating. A score of 0 was given to normal uptake in the lesion.

### Statistical analysis

Values are reported as the mean ± standard deviation (SD). Clinical characteristics of the patients were compared using Student’s *t*-test or analysis of variance. Statistical analyses with unpaired or paired 2-tailed *t*-tests or Pearson’s correlation coefficients were conducted. Based on the results from our preliminary study [16] and using the normal approximation algorithm of the Pearson chi-square test, the normal approximation algorithm of the Farrington-Manning test, and the Walters approximation algorithm of the Fisher exact probability method, the sample size result was obtained through reverse deduction and multiple loop calculations [26], with *N* = 6, nt = 3, nc = 3, and power = 0.8102. AIs between groups were performed using the Mann–Whitney *U*-test or Kruskal–Wallis test, followed by a post hoc test if required. Data were analyzed using SPSS (version 18.0; SPSS, Chicago, IL, USA). *P* < 0.05 was considered statistically significant.

## Results

### Participants

Sixteen patients (7.56 ± 4.28 years old; range: 2–16 years) participated in the study. We collected the clinical and follow-up data of all patients, including seizure history, semeiology, interictal EEG, SEEG (if indicated), neuroimaging, surgical area, and postoperative pathology. Pathological tissue confirmed FCD II, with FCD IIb in 14 patients (87.5%) and FCD IIa in 2 patients (12.5%). Lesions were localized to the frontal area in 5 patients, parietal area in 5 patients, the temporal area in 4 patients, the occipital area in 1 patient, and frontoparietal area in 1 patient.

The patients included in the present study were children or adolescents. Therefore, it was not possible to recruit age-matched controls. The best effort was made to recruit young adults as controls, and PET imaging data were used in a comparative analysis of parametric AI between the patients and controls. In total, 16 healthy individuals (23.80 ± 3.54 years old) were included. The demographic and clinical characteristics of the participants are summarized in Tables 1 and 2.

**Table 1:** Clinical demographics of FCD II patients and controls

Variable	FCD II patients (*n* = 16)	Control subjects (*n* = 16)	*P*
Age, years	7.56 ± 4.28	23.80 ± 3.54	0.00
Male, *N* (%)	7 (43.7)	10 (62.5)	0.289
Right handedness, *N* (%)	15 (93.8)	16 (100)	0.326
^18^F-SynVesT-1 SUV of centrum semiovale (ml/cm^3^)	1.35 ± 0.52	1.38 ± 0.35	0.918
Age of seizure onset, years	3.93 ± 2.78	-	-
Duration of epilepsy, years	2.88 ± 3.34	-	-
Number of antiepileptic drugs, *N* (range)	1.8 (1–3)	-	-

**Table 2:** Clinical and neuropathological information of FCD II patients

Patient	Age (years)/sex	Age at onset (years)	Duration (years)	Pathology	Location
1	10/F	2	8	FCD IIb	Right parietal lobe
2	2/F	1	0.5	FCD IIb	Right parietal lobe
3	6/M	6	0.33	FCD IIb	Left frontal lobe
4	10/M	8	2	FCD IIa	Left frontal lobe
5	2/M	1	1	FCD IIb	Left temporal lobe
6	5/F	2	3	FCD IIb	Left temporal lobe
7	16/M	6	10	FCD IIb	Left frontoparietal
8	4/M	1	3	FCD IIb	Right frontal lobe
9	11/F	1	10	FCD IIa	Right occipital lobe
10	3/F	3	0.1	FCD IIb	Left temporal lobe
11	9/M	8	1	FCD IIb	Left temporal lobe
12	15/F	13	2	FCD IIb	Left parietal lobe
13	8/F	7	0.25	FCD IIb	Left parietal lobe
14	10/F	8	2	FCD IIb	Left parietal lobe
15	5/F	3	2	FCD IIb	Left frontal lobe
16	5/M	4	1	FCD IIb	Left frontal lobe

### Injection parameters

The radiochemical purity of ^18^F-SynVesT-1 was greater than 99% (Supplemental Fig. S1). The injected activity dose of ^18^F-SynVesT-1 was 109 ± 52 MBq (range: 48–170 MBq) for the patients and 213 ± 23 MBq (range: 151–244 MBq) for the controls. The injected radioactivity of ^18^F-FDG was 109 ± 52 MBq for the patients. ^18^F-SynVesT-1 injections were well tolerated, with no subjective or objective adverse effects detected.

### Visual assessment of MRI and PET images

**Fig. 1:**
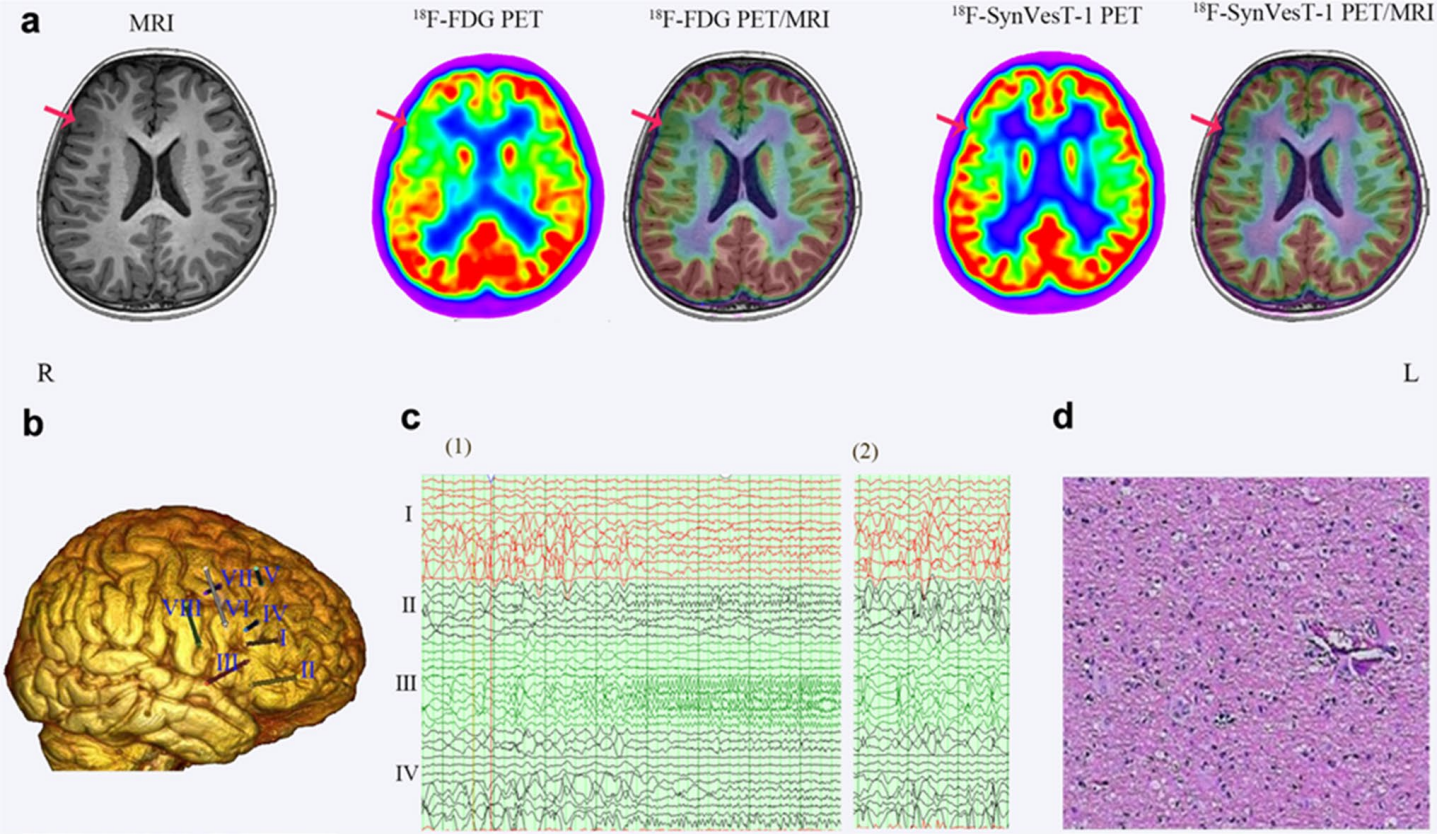
MRI, ^18^F-FDG, and ^18^F-SynVesT-1 PET/MRI images used for localization of the FCD II lesion in patient 8. **a** MRI image showed the thickening of the right inferior frontal gyrus; ^18^F-FDG PET and ^18^F-FDG PET/MRI images showed hypometabolism throughout the right inferior frontal gyrus and surrounding areas; ^18^F-SynVesT-1 PET and ^18^F-SynVesT-1 PET/MRI showed a more restricted area of low uptake in the frontalis inferior region (red arrows). **b** Overall electrode placement view. **c** Ictal (1) and postictal (2) SEEG recordings showed that the abnormal discharge area originated from the inferior frontal gyrus (II and III). **d** Postoperative pathology showed FCD IIb

**Fig. 2:**
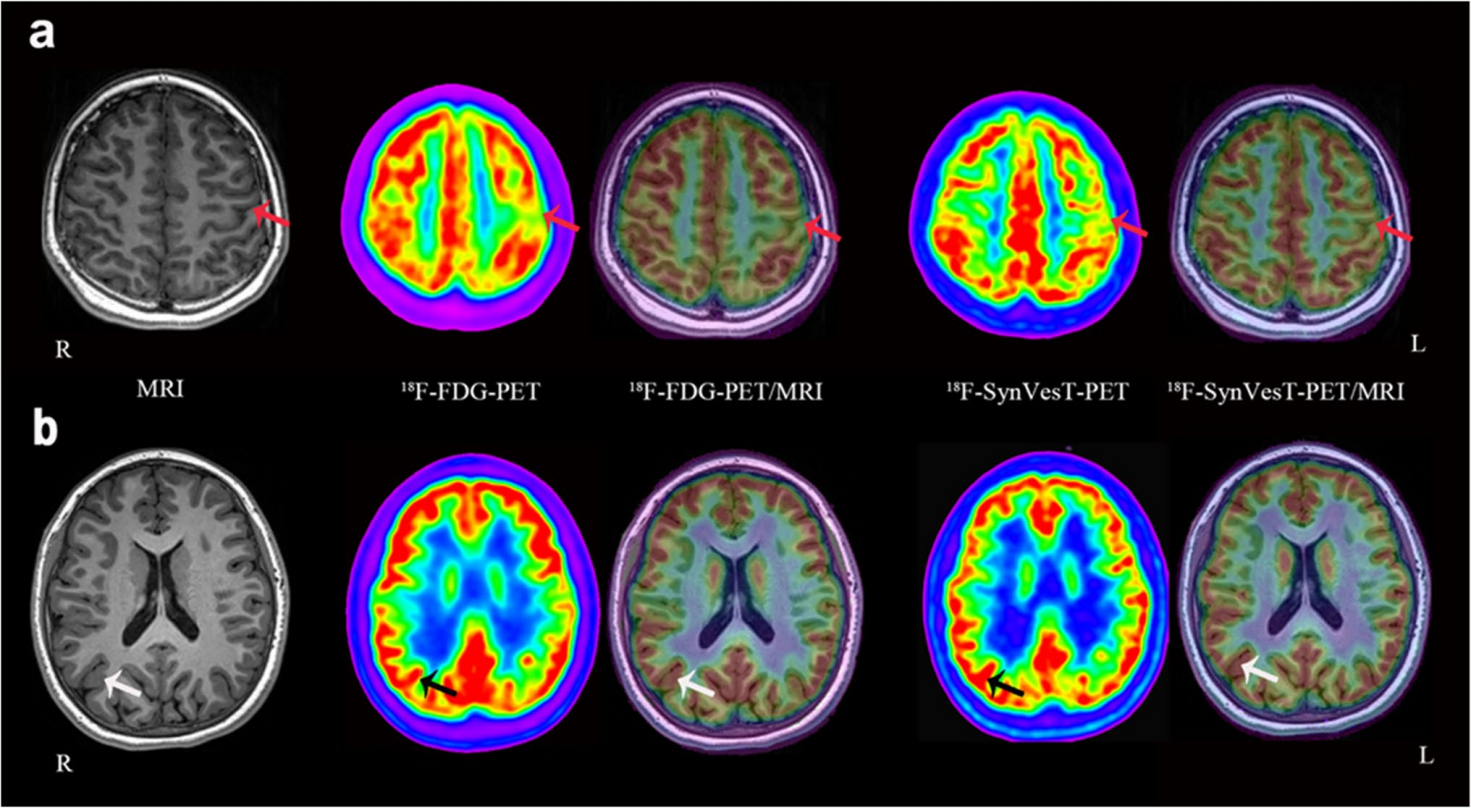
MRI, ^18^F-FDG, and ^18^F-SynVesT-1 PET/MRI images of the 2 FCD IIa patients. FCD IIa in two patients could not be detected by the initial visual assessment on ^18^F-SynVesT-1 PET. **a** The lesion in patient 4 was detected after superimposition of ^18^F-SynVesT-1 and ^18^F-FDG PET to MRI images, with the lesion located in the left frontal lobe (which displayed a small AI, 11.00%) (red arrows). **b** The lesion in patient 9 was not localized by the three neuroimaging methods. It was finally confirmed that the lesion was located in the right occipital lobe (white and black arrows). We assumed that FCD IIa might have a nonsignificant reduction in SV2A with ^18^F-SynVesT-1 PET

Visual assessment results are shown in Table 3. On review of the MRI images, lesions in 7 of 16 patients (43.8%) were correctly localized. ^18^F-FDG PET detected hypometabolism in 15 of 16 patients (93.8%). ^18^F-SynVesT-1 PET images showed low uptake in the FCD lesions compared with control images and nonlesioned areas of patients, with lesions correctly localized in 14 of 16 patients (87.5%) (Fig. 1). The localization rates were not significantly different between ^18^F-FDG and ^18^F-SynVesT-1 PET, and both were higher than the localization rate with MRI (*P* < *0.05*). ^18^F-SynVesT-1 PET SUV images showed false localization in 2 patients. One patient was found to have hypometabolism, but SV2A binding in the lesion was not significantly lower. The lesion was not localized by either SV2A or FDG PET/MRI images in only 1 patient (Fig. 2). Two patients were pathologically confirmed to have FCD IIa.

**Table 3:** Results of the visual assessments

	FCD II patients	Control subjects
MRI		
Positive	7/16 (43.8%)	0/16
Negative	9/16 (56.2%)	16/16 (100%)
^18^F-SynVesT-1 PET		
Positive	14/16 (87.5%)	0/16
Negative	2/16 (12.5%)	16/16 (100%)
^18^F-FDG PET		
Positive	15/16 (93.8%)	0/16
Negative	1/16 (6.2%)	16/16 (100%)
^18^F-SynVesT-1 PET/MRI		
Positive	15/16 (93.8%)	0/16
Negative	1/16 (6.2%)	16/16 (100%)
^18^F-FDG PET/MRI		
Positive	15/16 (93.8%)	0/16
Negative	1/16 (6.2%)	16/16 (100%)

### CS as a reference region

To ensure no between-group differences in nondisplaceable uptake for ^18^F-SynVesT-1, CS SUVs were compared and found to be nearly identical between controls (1.38 ± 0.35) and FCD II patients (1.36 ± 0.50) (*P* = 0.910), despite the age difference between the groups. Therefore, the CS appeared to be an appropriate reference region for the calculation of ^18^F-SynVesT-1 SUVr.

### Asymmetry in ^18^F-SynVesT-1 and ^18^F-FDG SUVr

Table 4 shows the results from the semiquantitative analyses of the PET data. The ^18^F-SynVesT-1 SUVr in the lesions in FCD II patients was significantly lower than the SUVr in the contralateral lobe (2.58 ± 0.97 vs. 3.67 ± 1.44, respectively, *P* < 0.05), while the SUVr difference between the nonlesioned ipsilateral and contralateral lobes was not significant (3.27 ± 1.25 vs. 3.50 ± 1.01, respectively, *P* = 0.58). The AI value with the SUVr was greater in the FCD II patients (27.14% ± 10.11%) than in the controls (2.4% ± 2.60%, *P* = 0.000). The AI values in the nonlesioned lobes showed no intersubject variability in either the FCD patients or controls (Table 5).

**Table 4:** Asymmetry index (AI) and SUVr of ^18^F-FDG and ^18^F-SynVesT-1 in the FCD lesion and nonlesioned lobes

		Lesion	Nonlesioned lobe	***P***
FDG	AI	27.08% ± 9.89%	5.06% ± 3.00%	0.00
	SUVr	2.82 ± 1.13	3.85 ± 1.33	0.02
SynVesT-1	AI	27.14% ± 10.11%	4.4% ± 2.30%	0.00
	SUVr	2.58 ± 0.97	3.67 ± 1.44	0.03

**Table 5:** Asymmetry indices for ^18^F-SynVesT-1 SUVr in the nonlesioned lobes of the patients and controls

	FCD II patients	Control subjects	***P***
Frontal lobe	2.1% ± 1.8%	2.1% ± 1.1%	0.918
Temporal lobe	2.4% ± 2.7%	3.6% ± 2.4%	0.23
Occipital lobe	2.4% ± 2.2%	2.6% ± 2.4%	0.724
Parietal lobe	3.7% ± 6.7%	3.0% ± 2.5%	0.767

^18^F-FDG uptake in the FCD II lesions was lower (SUVr of 2.82 ± 1.13) than that of the contralateral side (3.85 ± 1.33, *P* = 0.02). A significantly higher AI was observed in the patient group (27.08% ± 9.89%) than in the nonlesioned lobes (5.06% ± 3.00%, *P* < 0.05).

### Comparison of lesion asymmetry between ^18^F-FDG and ^18^F-SynVesT-1

As shown in Table 3, MRI was negative for lesion localization in 8 patients. After coregistration of ^18^F-FDG PET and MRI, the guided second reading changed the MRI report to a “subtle lesion” in 7 patients. The lesion AI based on ^18^F-SynVesT-1 uptake was not significantly different from that based on ^18^F-FDG uptake (27.14% ± 10.11% vs. 27.08% ± 9.89%, *P* = 0.841). There was a significant correlation (*R*^2^ = 0.41, *P* = 0.008) between the lesion AIs for ^18^F-SynVesT-1 SUVr and ^18^F-FDG SUVr in FCD patients (Supplemental Fig. S2).

### Visual ratings (^18^F-FDG PET versus ^18^F-SynVesT-1 PET)

**Fig. 3:**
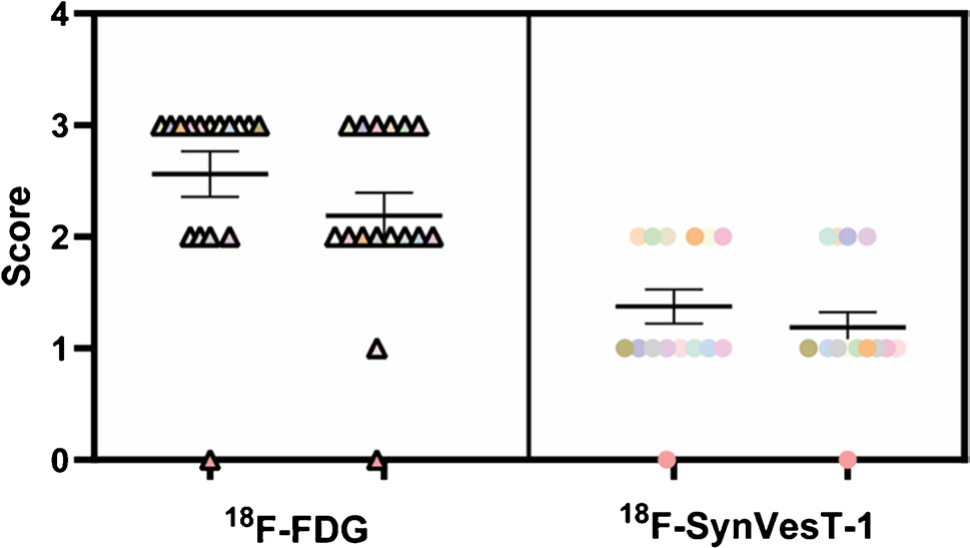
Grading of abnormal lesion uptake by two nuclear medicine specialists on the ^18^F-FDG and ^18^F-SynVesT-1 PET images. The abscissa represents the two nuclear medicine specialists and the two PET radioligands, the ordinate represents the score, and the same color points represent the same patient. On the ^18^F-SynVesT-1 PET images, the extent of decreased uptake was judged to be more restricted (1.281 ± 0.581) than that of ^18^F-FDG PET images (2.375 ± 0.832, *P* < 0.05)

In the initial visual assessment and semiquantitative analysis, we found no statistically significant difference between ^18^F-FDG and ^18^F-SynVesT-1 PET in the accuracy of lesion location and the AIs that reflected the intensity of decreased uptake in the lesion. In the subsequent visual rating of PET images, based on the extent of decreased lesion uptake, the pattern for ^18^F-SynVesT-1 was judged to be more restricted than that of ^18^F-FDG (score of 1.281 ± 0.581 vs. 2.375 ± 0.832, respectively, *P* < 0.05) (Fig. 3). This is consistent with the representative PET images shown in Fig. 1, where the area of decreased uptake for ^18^F-SynVesT-1 is narrower than that for ^18^F-FDG.

## Discussion

In the present study, we detected, for the first time, significantly lower SV2A binding in brain lesions of FCD II patients under the age of 18 using in vivo PET imaging with the novel SV2A radioligand ^18^F-SynVesT-1. Using visual and semiquantitative analyses of PET images coregistered with MRI images, we assessed the utility of ^18^F-SynVesT-1 compared with ^18^F-FDG PET for lesion localization in FCD II. Our results indicated that static ^18^F-SynVesT-1 PET provided high-quality images and that visual and semiquantitative analyses without arterial blood sampling enabled the identification of lesions in FCD II patients with accuracy comparable to the current standard static ^18^F-FDG PET. Furthermore, we demonstrated that the range of low SV2A binding in the lesions was more restricted than that of hypometabolism. The corresponding asymmetry in lesion uptake was highly correlated between the two radiotracers across subjects.

Lower SV2A in epileptic lesions has been repeatedly observed in brain tissue slices from animal models of epilepsy and epileptic patients [7, 8, 27]. In this study, we found an AI based on lesion SUVr of 27.14% ± 10.15% in patients with FCD II, which was much higher than that in the controls. This magnitude of SV2A reduction is in line with the reported 28% decrease for SV2A in FCD lesions measured in vitro with tissues resected from intractable epilepsy patients [10, 20]. Studies have suggested that the loss of SV2A may contribute to instability in neuronal networks [28]. Electrophysiological studies of spontaneous inhibitory neurotransmission revealed that the loss of SV2A led to an imbalance between glutamatergic and GABAergic neurotransmission [8, 29], thus implicating SV2A as a sensitive marker of FCD II, and PET imaging with SV2A-specific tracers as a potentially valuable tool for lesion identification/localization in this disorder. The comparison of lesion AI with ^18^F-SynVesT-1 SUVr and ^18^F-FDG SUVr indicated a positive correlation. One study in temporal lobe epilepsy also found that hippocampal ^18^F-FDG uptake was correlated with ^11^C-UCB-J binding potential [10]. These findings suggest that the hypometabolism in FCD II lesions may be related to the decrease in SV2A, and SV2A PET provides a complementary measure of the epileptogenic substrate.

We found that lesions in 2 FCD type IIa patients could not be detected by the initial visual assessment of ^18^F-SynVesT-1 PET images. In one patient, the lesion could be located after coregistration with MRI (and there was a small AI, 11.00%, in this patient). We assumed that FCD IIa might have resulted in a nonsignificant reduction in SV2A that was not clearly visible in the ^18^F-SynVesT-1 PET images but was associated with the pathological findings. Although cytological differences have indicated biological differences between the 2 FCD subtypes, no associated differences in clinical or imaging findings have been consistently identified. A previous study in FCD IIa showed decreased neuropil expression for SV2A, but strong perikaryal SV2A immunoreactivity has been observed around cytomegalic neurons [30]. The cytomegalic neuron expressed strong SV2A, which could obscure the originally decreased neuropil expression of FCD, and PET cannot differentiate these changes at the cellular level.

The detection of FCD II by MRI remains a challenge, despite the use of best-practice MRI sequences, new high-field MRI, and postprocessing software [5]. In the present study, MRI was negative in 56.2% of patients. ^18^F-FDG PET was reported to have a high sensitivity to detect FCD II in 60–92% of patients [6], and our ^18^F-FDG PET data were concordant with the findings from these contemporary studies. We found the visual assessments of SV2A PET images to have 87.5% accuracy in lesion localization, and there were no statistically significant differences between ^18^F-FDG and ^18^F-SynVesT-1 PET in the accuracy of lesion location (93.8% vs. 87.5%, respectively) and AI (27.14% ± 10.11% vs. 27.08% ± 9.89%). ^18^F-SynVesT-1 PET coregistered with MRI increased detection accuracy and helped delineate FCD II in MRI-negative/doubtful patients, with 93.8% accuracy in FCD detection.

More importantly, we demonstrated that ^18^F-SynVesT-1 PET images showed a more restricted pattern of reduced SV2A than the region of hypometabolism in FCD II patients. These findings may have practical importance, as in these cases, seizure freedom may be obtained by smaller resections that are precisely limited to the lesion. Hence, ^18^F-SynVesT-1 PET might be helpful to guide the placement of intracranial electrodes and presurgical planning. We assume that SEEG may remain indicated to limit the extent of resection, which is an important consideration when planning surgical resection in the eloquent cortex. However, such an invasive procedure may be progressively discontinued once multimodal imaging becomes capable of delineating the dysplastic cortex. Whether the more restricted pattern of reduced SV2A can become a real advantage for surgical planning of FCD patients requires more clinical research and data analysis in the future.

^18^F-SynVesT-1 has been shown to have excellent kinetic and in vivo binding properties in the human brain. SUVr from 60–90 min postinjection provided a good match with the one-tissue compartment binding potential of ^18^F-SynVesT-1 and can serve as a surrogate quantitative measurement of specific binding in a short scan time without invasive arterial sampling [15]. As it is more difficult for younger patients with FCD to maintain the same posture for a long time, the longer half-life of ^18^F coupled with a quantitative measurement in a short scan time with ^18^F-SynVesT-1 will facilitate its broad application in studies of SV2A in FCD and other neuropsychiatric populations.

There are several limitations that warrant mentioning. First, our sample size was modest, although it is well within the range for a PET study evaluating a new radiotracer in a new patient group such as FCD patients [6]. The limited sample size may have obscured possible correlations with clinical measures. Second, we used the CS as a reference region to adjust for nonspecific uptake in the brain. However, it may not be the optimal reference area because its tissue composition is different from gray matter, and the use of the CS as a reference region might lead to underestimation of the SUVr [31]. Last, the age difference between the patient and control groups might have had an effect on the study findings. We analyzed the SUVr of nonpathological brain regions for all participants to observe the trend in synaptic density changes (Supplemental Fig. S3), which was consistent with results from previous molecular biology studies on synaptic pruning and synaptic density changes with age [32, 33]. The changes in myelination with age is another factor that needs to be considered [34]. Postmortem and neuroimaging studies have suggested a quadratic relationship between myelination status and age, which might impact synaptic changes [35, 36]. Due to the age difference between the two groups, objective analyses, such as statistical parametric mapping (SPM), could not be conducted. As a result, we performed only a relatively subjective visual assessment and semiquantitative analysis/comparison between the child patient group and young adult control group. Further analysis will be conducted in future in-depth studies as we continue our investigation in this patient population.

## Conclusions

To the best of our knowledge, this is the first in vivo study to investigate SV2A in the lesions of living people with FCD II by PET imaging with the radioligand ^18^F-SynVesT-1. ^18^F-SynVesT-1 PET demonstrated a higher accuracy than MRI for the localization of FCD II lesions, with a more restricted pattern of SV2A abnormality than that of hypometabolism detected by ^18^F-FDG PET. In conclusion, SV2A PET imaging may provide a more specific localization of lesions in FCD II, and in presurgical evaluation and planning, it can serve as a complementary measure of the epileptogenic substrate in addition to the established clinical assessments.

## Supplementary Information

Below is the link to the electronic supplementary material.

Supplementary file1 (DOCX 299 KB)

## Data Availability

The datasets generated and/or analyzed during the current study are available from the corresponding author on reasonable request.
