# Supplementary material for: Cortical abnormalities of synaptic vesicle protein 2A in focal cortical dysplasia type II identified in vivo with 18F-SynVesT-1 positron emission tomography imaging
Source: Eur J Nucl Med Mol Imaging. 2022 Jan 3;49(10):3482–91. doi: 10.1007/s00259-021-05665-w (PMC9308579; doi:10.1007/s00259-021-05665-w)
Supplement: Supplementary file 1 — Supplementary file1 (DOCX 299 KB) [file 259_2021_5665_MOESM1_ESM.docx]

**Supplementary Information**

**Article title:** Cortical abnormalities of synaptic vesicle protein 2A in focal cortical dysplasia type II identified *in vivo* with ^18^F-SynVesT-1 positron emission tomography imaging

**Journal name:** European Journal of Nuclear Medicine and Molecular Imaging

**Author names:** Yongxiang Tang^1^, Jie Yu^1^, Ming Zhou^1^, Jian Li^1^, Tingting Long^1^, Yulai Li^1^, Li Feng^2^, Dengming Chen^1^, Zhiquan Yang^3^, Yiyun Huang^4^, Shuo Hu^1, 5, 6^

**Affiliations:**

^1^ Department of Nuclear Medicine, Xiangya Hospital, Central South University, Changsha, Hunan, China.

^2^ Department of Neurology, Xiangya Hospital, Central South University, Changsha, Hunan, China.

^3^ Department of Neurosurgery, Xiangya Hospital, Central South University, Changsha, Hunan, China.

^4^ PET Center, Department of Radiology and Biomedical Imaging, Yale University School of Medicine, New Haven, CT, USA

^5^ Key Laboratory of Biological Nanotechnology of National Health Commission, Xiangya Hospital, Central South University, Changsha, Hunan, China.

^6^ National Clinical Research Center for Geriatric Disorders (XIANGYA), Xiangya Hospital, Central South University, Changsha, Hunan, China.

**E-mail address of the corresponding author:**

Shuo Hu, [hushuo2018@163.com](mailto:hushuo2018@163.com); Yiyun Huang, henry.huang@yale.edu

**Fig. S1 HPLC chromatogram from co-injection of ^18^F- and ^19^F-SynVesT-1.**

**Fig. S2** **Correlation between asymmetry index measured by standardized uptake value ratios of ^18^F-SynVesT-1 and ^18^F-FDG in lesions of the patients.**

**Fig. S3 Change of regional ^18^F-SynVesT-1 standardized uptake value ratio with age.**


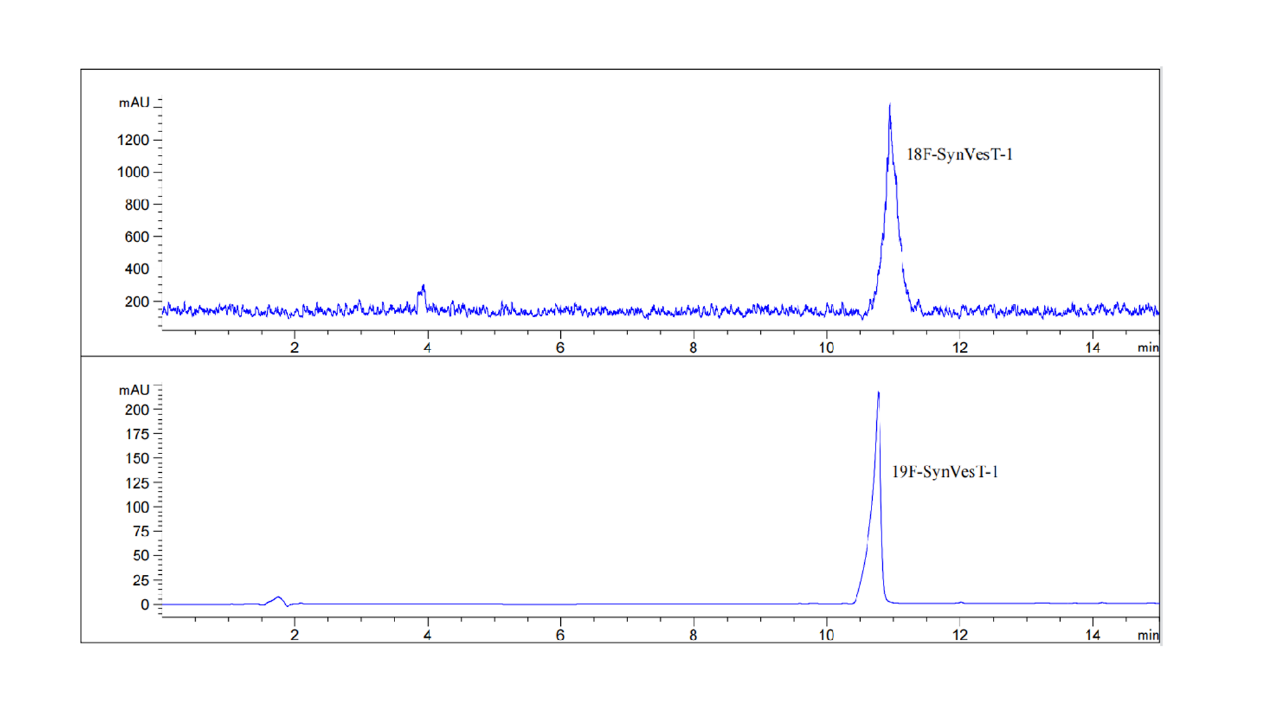
 **Fig. S1** **HPLC chromatogram from co-injection of ^18^F- and ^19^F-SynVesT-1**

^18^F-SynVesT-1 specific activity: 308.3MBq/nmol. The radiochemical purity was greater than 99%.


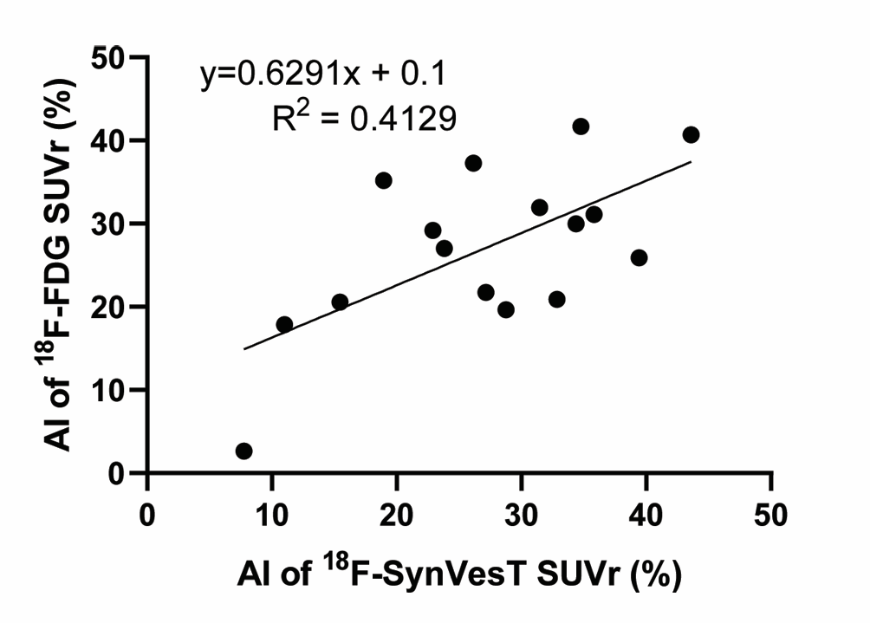


**Fig. S2 Correlation between asymmetry index measured by standardized uptake value ratio of ^18^F-SynVesT-1 and ^18^F-FDG in lesions of the patients**
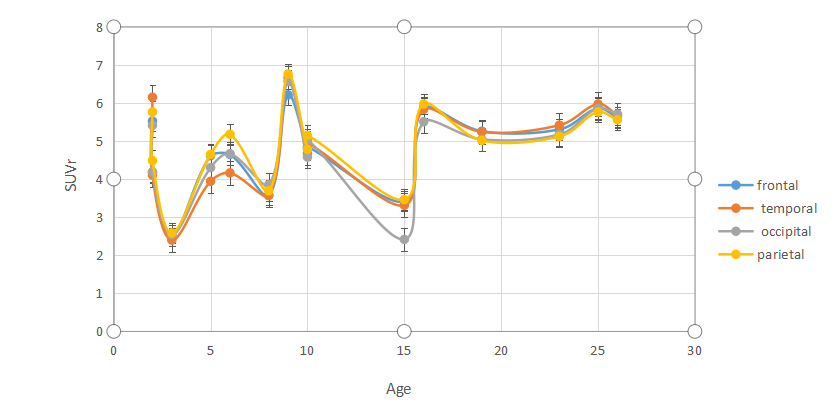


**Fig. S3** **^18^F-SynVesT-1 standardized uptake value ratio-age curves in the no lesion brain regions of FCD patients aged 2–16 years and in normal brain regions of controls aged 19–26 years**

^18^F-SynVesT-1 PET scanning *in vivo* showed SUVr of each non-lesion lobes and synaptic density changes with age 2–16 years, and in normal brain lobes of controls age 19–26 years. SUVr-age curves varied widely in non-lesion brain regions of FCDII patients aged 2–16 years, but the curves were relatively stable in brain regions of the young adult control group.
